# Gene expression, evolution, and the genetics of electrosensing in the smalltooth sawfish, *Pristis pectinata*

**DOI:** 10.1002/ece3.11260

**Published:** 2024-04-29

**Authors:** Taiya M. Jarva, Nicole M. Phillips, Cory Von Eiff, Gregg R. Poulakis, Gavin Naylor, Kevin A. Feldheim, Alex S. Flynt

**Affiliations:** ^1^ School of Biological, Environmental, and Earth Sciences The University of Southern Mississippi Hattiesburg Mississippi USA; ^2^ Charlotte Harbor Field Laboratory Fish and Wildlife Research Institute, Florida Fish and Wildlife Conservation Commission Port Charlotte Florida USA; ^3^ Florida Program for Shark Research University of Florida Gainesville Florida USA; ^4^ Pritzker Laboratory for Molecular Systematics and Evolution, the Field Museum Chicago Illinois USA

**Keywords:** conservation, electrosensing, evolution, sawfish, transcriptome

## Abstract

Sawfishes (Pristidae) are large, highly threatened rays named for their tooth‐studded rostrum, which is used for prey sensing and capture. Of all five species, the smalltooth sawfish, *Pristis pectinata*, has experienced the greatest decline in range, currently found in only ~20% of its historic range. To better understand the genetic underpinnings of these taxonomically and morphologically unique animals, we collected transcriptomic data from several tissue types, mapped them to the recently completed reference genome, and contrasted the patterns observed with comparable data from other elasmobranchs. Evidence of positive selection was detected in 79 genes in *P. pectinata*, several of which are involved in growth factor/receptor tyrosine kinase signaling and body symmetry and may be related to the unique morphology of sawfishes. Changes in these genes may impact cellular responses to environmental conditions such as temperature, dissolved oxygen, and salinity. Data acquired also allow for examination of the molecular components of *P. pectinata* electrosensory systems, which are highly developed in sawfishes and have likely been influential in their evolutionary success.

## Graphical abstract

Sawfishes (Pristidae) are large, highly threatened rays named for their tooth‐studded rostrum, which is used for prey sensing and capture. To better understand the genetic underpinnings of these unique animals, we used RNA‐seq data from multiple tissues to annotate a publicly available genome and compared this data to other elasmobranchs. Evidence of unique positive selection was seen in Pristis pectinata in genes related to growth factor signaling and organ symmetry, suggesting a role in morphogenesis. Data acquired also allowed examination of electrosensory components.

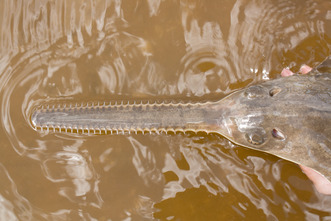

## 1 INTRODUCTION

As meso‐ to apex‐level predators, chondrichthyans (sharks, rays, and chimaeras) play essential roles in the health of marine ecosystems by increasing biodiversity, buffering against invasive species, decreasing transmission of diseases, and mitigating the effects of climate change (Ferretti et al., 2010; Ritchie et al., 2012). However, many chondrichthyans are in decline, primarily due to overfishing, with more than one third of species estimated to be threatened with extinction (Dulvy et al., 2021). Sawfishes belong to one of the most threatened families, with all five species assessed as Endangered or Critically Endangered on the International Union for the Conservation of Nature (IUCN) Red List of Threatened Species (D'Anastasi et al., 2013; Carlson et al., 2022; Espinosa et al., 2022; Grant et al., 2022; Harry et al., 2022).

Sawfishes are notable for their tooth‐studded rostrum, which is used to detect, acquire, and manipulate prey (Poulakis et al., 2017; Wueringer, 2012). However, this rostrum also makes them prone to entanglement in fishing gear, which has precipitated their declines over the last century (Dulvy et al., 2016). Bycatch in fisheries and habitat degradation continue to pose the greatest threats, including in ‘stronghold’ locations such as the southeastern United States and western and northern Australia (Brame et al., 2019; Yan et al., 2021). Fisher education and trade bans on sawfishes and their parts have been used to mitigate mortalities in fisheries (Wiley & Brame, 2018). While such strategies are essential, additional preventative initiatives are needed as sawfishes continue to interact with fisheries globally (Yan et al., 2021).

Chondrichthyans are well known for their ability to sense prey through their bioelectric signature, and it has been suggested that this feature can be used to design behavioral interventions to reduce bycatch (Abrantes et al., 2021; Fields, 2007). Initial sensing occurs through voltage‐gated calcium channels (VGCC) produced from *Cacnα1D* in both rays and sharks (Bellono et al., 2017). Following influx of Ca^2+^ ions, K^+^ efflux occurs leading to membrane depolarization and neurotransmission. Different K^+^ channels participate in rays (BK channels, BK‐α) and sharks (Shaker‐type, Kv) (Bellono et al., 2018). Sawfishes have large numbers ampullae of Lorenzini distributed along the entire length of their rostrum, allowing for heightened electroreception abilities compared to other chondrichthyans (Wueringer, 2012; Wueringer et al., 2011). Recent research aimed at supporting the development of deterrent technology that exploits this highly sensitive electrosensory capability found that largetooth sawfish, *Pristis pristis*, reacted to electric field stimuli, but behavioral responses were not consistent and were considered insufficient to avoid fisheries gear entanglement (Abrantes et al., 2021). Having a better understanding of the underlying molecular mechanisms of electroreception and biosensing in sawfishes could reveal molecular‐level responses to external stimuli, supporting these behavioral studies.

The aim of this study was to explore the genetics of the smalltooth sawfish, *Pristis pectinata*, to identify physiological features that might impact conservation approaches. *Pristis pectinata* was specifically targeted because compared to other sawfishes, this species has experienced a great decline in range, being present in less than 20% of its former range in the Atlantic Ocean (Dulvy et al., 2016). Viable populations are currently restricted to Florida in the U.S. and western portions of The Bahamas and, like all sawfishes, reducing fisheries interactions is a top conservation priority (Carlson et al., 2022).

To study *P. pectinata* genetics, RNA sequencing data from different tissues were acquired and used in conjunction with a high‐quality, publicly available genome assembly to generate a near‐complete gene set. From this, patterns of gene expression and sequence evolution were compared to those from other elasmobranchs to identify unique genetic features. Analysis of the gene set also permitted characterization of the molecular features of putative *P. pectinata* electrosensing components, which may be targeted to affect behavior. Thus, this investigation of genetic evolution in *P. pectinata* will serve as a foundation for future work focused on physiology, interactions with the environment, and impacts of stressors on the health, growth, and recovery of sawfishes.

## 2 METHODS

### 2.1 RNAseq‐assisted genome annotation

**FIGURE 1:**
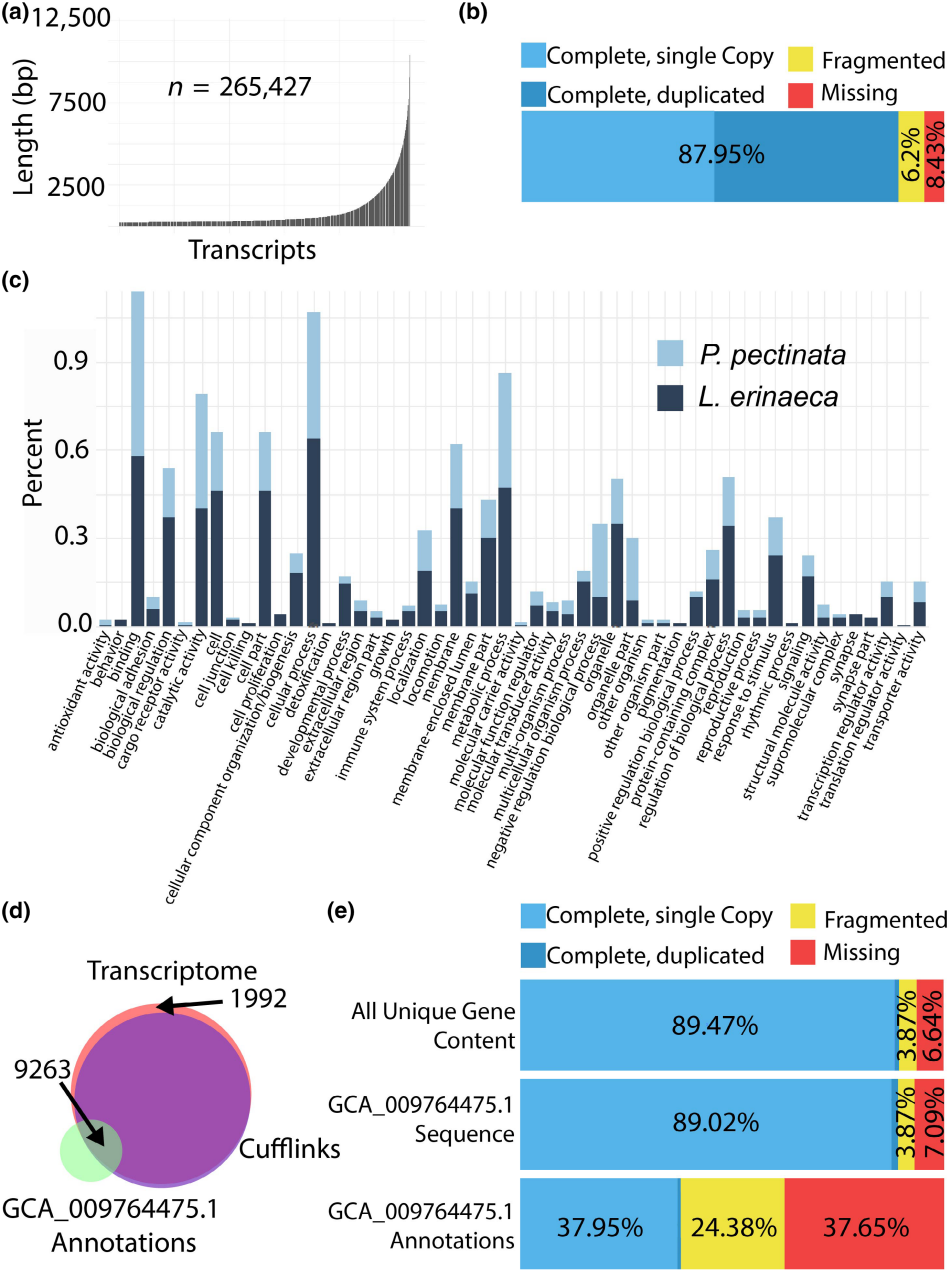
Establishment of a near‐complete gene set for the smalltooth sawfish, *Pristis pectinata*. (a) Log length distribution of transcripts in assembled transcriptome with total number of transcripts. (b) Outcome of Benchmarking Universal Single Copy Ortholog (BUSCO) analysis to assess completeness of assembled transcriptome. (c) WeGO (Ye et al., 2018) gene ontology analysis of *P. pectinata* and little skate, *Leucoraja erinacea*, transcriptomes. Genes are classified according to biological process, molecular function, or cellular component and plotted by percent of genes related to meaningful biological activities. (d) Venn diagram (Hulsen et al., 2008) showing overlap of the number of transcripts provided with the public genome (GCA_009764475.1, green), the Cufflinks (purple) assisted annotation of the public genome, and transcripts in the de novo assembled transcriptome (pink). (e) BUSCO assessment for all unique gene content from combined transcriptome and genome resources, and annotations associated with GCA_009764475.1.

The Vertebrate Genome Project has released a high‐quality reference genome assembled from an adult female *P. pectinata* (GCA_009764475.1, http://vertebrategenomesproject.org). While the assembly had high contiguity, there were limited expressed sequence tags (EST) data associated with the original genome assembly, which results in significant gene annotation challenges (Salzberg, 2019). As a result, approximately 40% of expected orthologs were absent from the predicted gene dataset (Figure 1) (Simão et al., 2015). RNA‐seq data in conjunction with a high‐quality genome assembly can be used to capture confident exons for better construction of open reading frames. Thus, to maximize the utility of the genome, RNA was sequenced from tissues collected from a juvenile female (828 mm stretch total length) collected under Endangered Species Act (ESA) Permit No. 21043.

### 2.2 Sample collection for transcriptome sequencing

RNAs were collected from the brain, liver, kidneys, ovary, and skin tissues of a juvenile female *P. pectinata*. Tissues were preserved in RNAlater (Thermo Fisher Scientific, Waltham, MA) and stored at −80°C until use. Samples from each tissue were homogenized and extracted with the TRIzol method. Approximately 100 mg from each tissue type were homogenized and extracted with 1 mL TRIzol (Thermo Fisher Scientific) and incubated at 20°C for 10 min, followed by clarification by centrifugation. Supernatant was combined with chloroform and phase separated. The aqueous phase was precipitated in isopropanol, the pellet was washed in 70% ethanol, and suspended in nuclease‐free water. Concentrations and purities of RNA extracts were measured using a Nanodrop (Thermo Fisher Scientific) and a Bioanalyzer 2100 (Agilent, Santa Clara, CA) (Table S1). Unstranded libraries were prepared by Novogene® using a TruSeq RNA Prep Kit v2 (Illumina, San Diego, CA), which uses polyA purification followed by fragmentation, random‐primed reverse transcription, Y‐adapter ligation, and PCR. 20 M reads were generated per sample using a paired‐end 150 base pair protocol.

### 2.3 Transcriptome sequencing and assembly

The libraries were sequenced by Novogene® and the transcriptome was assembled using Trinity‐v2.5.1 on the Magnolia High Performance Computing (HPC) cluster (Grabherr et al., 2011). Adapters were removed with CutAdapt v1.8 and all read pairs containing “N” bases were discarded. Read quality during alignment was filtered by SAMtools v1.13 using a MAPQ cut off of 10 (Li et al., 2009).

Blast2GO was used to assign gene ontology terms to transcripts using Phylum Chordata (NCBI taxa 7711) as the query database, and WeGO was used for comparison between *P. pectinata* and the little skate, *Leucoraja erinacea* (Gotz et al., 2008; Ye et al., 2018). Completeness was assessed with Benchmarking Universal Single Copy Ortholog (BUSCO) in transcriptome mode against the most recent vertebrate lineage, odb10 (BUSCO v1.4.33). To annotate the genome with coding sequences and to capture a more complete gene set for positive selection analysis, the published *P. pectinata* genome from an adult female (GCA_009764475.1) was used in addition to the assembled transcriptome.

### 2.4 Gene content for positive selection analysis

Gene content exclusive to the transcriptome was isolated using STAR aligner v2.7.7 with default parameters to align reads from all tissue samples to the genome (Dobin et al., 2013). Coverage over the genome was assessed with BEDtools v2.30.0 and regions with low coverage (<5 reads) were then aligned to the assembled transcriptome using Hisat2 v2.2.1 with the –dta‐cufflinks option enabled (Kim et al., 2015; Quinlan & Hall, 2010). Cufflinks v2.2.1 was used to map RNA reads to coordinates in the genome, which were then intersected, excluding overlapping transcripts, with Augustus‐predicted gene sequence coordinates for the genome using BEDTools Intersect (Trapnell et al., 2010). All BAM and SAM file conversion and sorting was performed with SAMtools v1.13 (Li et al., 2009). Unique gene sequences from the transcriptome and genome were concatenated into one dataset for further analysis and BUSCO annotation output was used to remove redundant sequences and assess completeness. To obtain transcript expression, each tissue library was individually mapped to the constructed transcriptome using Bowtie2 v2.4.4, and SAMtools Idxstats was used to quantify mapped reads (Kim et al., 2015). Annotation of GCA_009764475.1 using RNA seq is available (Appendix S1).

### 2.5 Positive selection in transcriptome

Transcriptomes from smalltooth sawfish and four other fish species, Australian ghostshark, *Callorhinchus milii*, chain catshark, *Scyliorhinus retifer*, little skate, *L. erinacea*, and the Indonesian coelacanth, *Latimeria menadoensis*, were used to predict open reading frames using TransDecoder v3.0.1 with default parameters (Hass, n.d.). All coding sequences were concatenated into one dataset and OrthoFinder was executed with default parameters to cluster orthologous gene groups (Emms & Kelly, 2015). Sequences in each gene family were annotated using eggNOG with Diamond mode enabled (Huerta‐Cepas et al., 2017). Annotations were also used to split gene families into paralogous groups using custom scripts. For positive selection, only groups that retained at least one sequence from each taxon were retained for analysis. Transcripts were discarded if their length was more than 100 amino acids shorter or longer than the average length of the gene, and only genes with Pal2Nal alignments longer than 20 amino acids were kept, excluding trees with insufficient branch lengths for analysis. aBSREL, which identifies episodic selection in individual branches, was used to analyze each group of orthologous genes (Smith et al., 2015). Mafft v7.475 was used for protein alignment, FastTree 2.1.10 for tree construction, and Pal2Nal v14 with the –nogap option to provide gap‐free codon‐based nucleotide alignments (Katoh & Standley, 2013; Price et al., 2009; Suyama et al., 2006). Genes with Holm‐Bonferroni corrected *p*‐values ≤.05 and uniquely under selection in *P. pectinata* were found by parsing output JSON files with custom scripts using the JsonLite package (Ooms, 2014). The repository containing the parse aBSREL selection and all pipelining scripts can be found at https://github.com/stupornova33.

To examine potential effects of substitutions in genes of interest under selection, chemical properties were compared between protein sequences from *P. pectinata* and *L. erinacea* using Expasy ProtScale (Gasteiger et al., 2005). The Kyte & Doolittle (Kyte & Doolittle, 1982) scale was used for hydrophobicity and the Zimmerman scale for polarity (Zimmerman et al., 1968). After manual gap correction, scales at each residue for *P. pectinata* were subtracted from *L. erinacea* values and the change plotted by residue. Domains of each protein were obtained from InterProScan. A proportion of PSGs had omega values >10,000, indicating the presence of non‐synonymous substitutions and a lack of synonymous substitutions. Thus, analysis of PSGs of interest using DAMBE found no significant substitution saturation in any species from any alignment, indicating that the species are not too diverged to obtain meaningful positive selection results (Xia, 2018; Appendix S2). Additionally, the use of codon‐aligned sequences with the “–no‐gaps” option enabled ensured that selection tests were not performed on diverged regions.

For principal components analysis (PCA) analysis, percentage of sites and omega values were taken from aBSREL analysis described above. Grand average of hydropathy (GRAVY) values for each *P. pectinata* and *L. erinacea* protein were obtained from Expasy ProtParam. If there were two sequences in an orthogroup for *L. erinacea*, the closest aligning sequence was selected for each *P. pectinata* gene from a multiple sequence alignment, and the difference was taken between the values. Principal component analysis was performed using the factoextra R package v1.0.7 and visualized with ggpubr v0.6.0 (Kassambra, 2020) (Figure S2).

### 2.6 Conservation of changes in sawfishes

To determine whether signals of positive selection in genes of interest for *P. pectinata* were also present in other sawfishes, nucleotide changes in the three other *Pristis* sawfishes were examined for *HoxA5* and *Ccdc103*. Primers were designed to amplify divergent homologous functional domains between *P. pectinata* and *L. erinacea*. Primer sequences were: *HoxA5* forward: 5′‐GACTTATGTGCAGTTTTCGCATCCA‐3′; *HoxA5* reverse: 5′‐AACTACCTCCTCAAATTC‐3′; *Ccdc103* forward: 5′‐CTGCTGCTCAGGAAATCCAC‐3′; *Ccdc103* reverse: 5′‐AGCGGAGTTTAGCCGTGACTG‐3′.

DNA extracts from *P. clavata*, *P. zijsron*, and *P. pristis* were mixed with Phire Hot Start II DNA polymerase (Thermo Fisher Scientific), dNTPs, UltraPure DNase/RNase‐Free Distilled water (Thermo Fisher Scientific), and primers (Eurofins) for either *Ccdc103* or *HoxA5*, followed by PCR amplification using a Mastercycler Pro and electrophoresis apparatus. Concentrations of the PCR reactions were 1× Phire Reaction Buffer, 80 μM dNTPs, 0.5 μM of each of a forward and reverse primers (listed above), and 25 ng/μL genomic DNA. The reactions were initially denatured at 98°C for 30 s followed by 35 cycles of: 98°C for 5 s, 53°C (*HoxA5*) or 58.5°C (*Ccdc103*) for 15 s, and 72°C for 1 min. PCR reactions for each locus ended with a final extension of 1 min.

PCR products were purified following 1.5% standard molecular grade agarose gel (Fisher BioReagents) for 35 min at 125 V. The bands were excised and purified with the GeneJET Purification Kit (Thermo Fisher Scientific) and sent to Eurofins Genomics for sequencing. ApE (RRID:SCR_014266) was used to translate DNA sequences into the appropriate reading frame, and Clustal Omega was used to align sequences to *P. pectinata* and *L. erinacea* (Madeira et al., 2022). BLAT from the UCSC genome browser and the *P. pectinata* genome were used to verify amplification of the correct target sequence. Alignments were manually examined between species to identify conserved non‐synonymous changes among sawfishes that were not found in *L. erinacea*.

### 2.7 Electrosensory genes

To identify *P*. pectinata electrosensory genes, full coding sequences for *L. erinacea* electrosensing genes were downloaded from NCBI (acc. AJP74816.1, KY355736.1) as query and used to BLAST against a protein database which was constructed from the sawfish transcriptome by Hmmer2GO (Staton, 2018). Analysis included the VGCC (*Cacnα1D*) and several β‐subunits, potassium‐activated (BK) channel α‐subunit, and several Shaker (Kv) channels which have been implicated as major ion channels involved in electroreception in elasmobranchs (Bellono et al., 2017). BK*β* and Shaker‐type channels were identified using BLAST and confirmed using InterProScan (Jones et al., 2014). Alignments were performed using ClustalW with default parameters and 1000 bootstraps and visualized using the GGMsa v.1.0.3 package in R (Zhou et al., 2022). The phylogenetic tree was visualized with the GGTree package v3.4.2 (Yu et al., 2017). GenBank accessions for the elasmobranch sequences most similar to the uncharacterized BK*β* subunit were: XP_032892500.1 (*Amblyraja radiata*), GCB66272.1 (*Scyliorhinus torazame*), XP_038668782.1 (Scyliorhinus canicula), and XP_043564107.1 (Chiloscyllium plagiosum) (accessed 26 September 2022).

## 3 RESULTS

Genome annotation facilitated by RNA‐seq alignments yielded substantially more transcripts (159,014), than the 19,597 genes predicted from the genome alone (Appendix S1). RNA‐seq data were also assembled into a de novo transcriptome of 265,427 transcripts with 31.9% being >500 bp (Figure 1a,b). Additional improvement to the collection of *P. pectinata* genes gained by RNA‐seq guided annotation came from a de novo transcriptome that had ~2000 genes not found in genome annotations–either from Augustus or Cufflinks (Figure 1d). Combining the RNA‐seq assisted annotations, the de novo transcriptome, and the reference genome assembly predictions resulted in a greatly enhanced gene set representation (Figure 1e). In the de novo transcriptome alone, less than 10% of expected orthologs, determined by BUSCO, were absent relative to the genome de novo annotation, where ~37% were missing. In the combined gene set, the percentage of missing genes was reduced to only 6.6% (Table S2).

To further validate the *P. pectinata* gene set, gene ontology (GO) terms were assigned and compared to the transcriptome of *L. erinacea* (Wyffels et al., 2014) (Figure 1c). Distributions of high‐level GO terms were similar between *P. pectinata* and *L. erinacea*, suggesting gene content in the de novo transcriptome represents what is observed in related taxa. However, differences were noted, such as a higher percentage of genes involved in antioxidant activity and DNA binding in *P. pectinata* versus a higher percentage of genes in *L. erinacea* related to signaling, response to stimulus, metabolic process, and catalytic activity. This may reflect a difference in juvenile and adult tissues sampled for *P. pectinata* relative to the embryonic tissue‐derived *L. erinacea* transcriptome. Another reason could be the more complete dataset for *P. pectinata* provided here versus *L. erinacea* (Table S2). The near‐complete *P. pectinata* gene set enables characterization of unique genetics in this species, which was not possible with predicted annotations offered by genome sequence approaches alone.

### 3.1 Unique genetic features of *Pristis pectinata*

**FIGURE 2:**
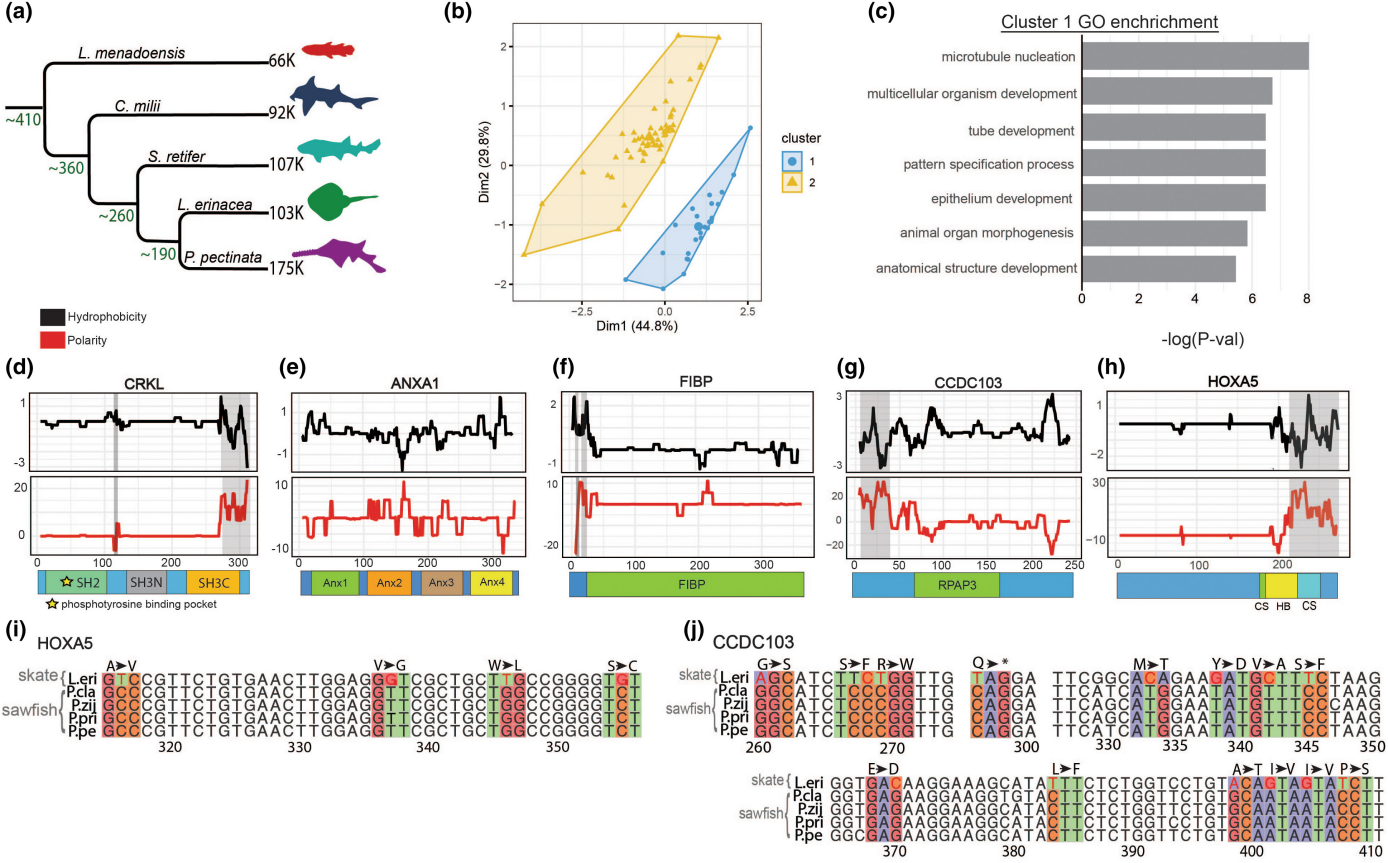
Smalltooth sawfish, *Pristis pectinata*, genes under positive selection. (a) Phylogeny of taxa used in positive selection analysis with approximate divergence times shown as millions of years ago at each node and number of transcripts in each transcriptome noted at each terminal branch. Species included: *Latimeria menadoensis*, *Callorhinchus milii*, *Scyliorhinus retifer*, *Leucoraja erinacea*, and *P. pectinata*. (b) Results of k‐means clustering analysis of genes with a percentage of sites under selection using omega value, percent of sites, change in grand average of hydropathy value relative to little skate, *L. erinacea*. (c) Enriched gene ontology (GO) terms related to biological processes from genes which were grouped into Cluster 1 by principal components analysis plotted by ‐log(*p*‐value). (d–h) Changes in hydrophobicity and polarity of protein sequence relative to *L. erinacea*. Sequence alignment gaps are shown by shaded regions. Functional protein domains retrieved from InterproScan and literature are shown as colored boxes below each plot. (i) Sequence alignment of PCR‐amplified region of interest from HoxA5 between *Pristis* sawfishes and *L. erinacea* with codons of interest colored by nucleotide. (j) Alignment of region of interest from amplification of CCDC103. Alignments are colored by nucleotide.

To identify genes that exhibit some proportion of sites experiencing positive selection, hereafter referred to as positively selected genes (PSGs) in *P. pectinata*, the combined dataset from *P. pectinata* and transcriptomes of the Australian ghostshark, chain catshark, little skate, and Indonesian coelacanth were assigned to orthologous gene groups with Orthofinder (Emms & Kelly, 2015) (Figure 2a). *Pristis pectinata* had the second highest percentage of genes (~51%) assigned to an orthogroup and the highest percentage of species‐specific genes, likely due to the substantially greater completeness of the assembly relative to the other species (Figure S1). The resulting 3116 genes were tested for branch‐specific episodic selection using aBSREL. This tool was used as it can detect short bursts of positive selection on a few sites in a lineage (episodic selection), which is often seen in evolutionary diversification (Cicconardi et al., 2017). From this, 79 PSGs were identified in *P. pectinata* (Smith et al., 2015) (Appendix S2: Methods; Table S3).

To cluster *P. pectinata* PSGs into groups which may share functional relationships or similar selection pressures, omega values from aBSREL, percentage of sites under selection, and the difference in GRAVY between *P. pectinata* and *L. erinacea* orthologs were collected for a PCA (Figure S2). K‐means clustering identified two groups containing 52 and 22 genes (Figure 2b & Figure S2a). Gene ontology enrichment analysis by TopGO v2.4.2 of PSGs in cluster 1 versus all genes analyzed showed enrichment in functions such as multicellular organism development, animal organ morphogenesis, and anatomical structure morphogenesis (Alexa & Rahnenfuhrer, 2022) (Figure 2c). In comparison, cluster 2 genes were related to transmembrane transport, proteolysis, and response to external stimulus (Figure S2b). Interestingly, cluster 1 contained multiple genes implicated in developmental processes including genes involved in fibroblast growth factor/receptor tyrosine kinase (FGF/RTK) and mitogen‐activated protein kinase (MAPK) signaling, reinforcing that clustered *P. pectinata* PSGs share functional or evolutionary similarities.

Comparing biochemical properties such as hydrophobicity and polarity of developmental genes with the closest available taxon, *L. erinacea*, revealed potentially functional changes in *P. pectinata* in three notable genes: *Crk‐l*, an integrator of multiple signaling pathways; Annexin A1 (*Anxa1*), a modulator of FGF ligands and RAS, and FGF1 intracellular binding protein (*Fibp*), an EGF/MAPK modulator (Figure 2d–h) (Balasubramanian & Zhang, 2016; Katoh & Katoh, 2006). Another signaling‐related gene is *Ccdc103*, which is necessary for ciliogenesis (Panizzi et al., 2012). CCDC103 had numerous changes in polarity and hydrophobicity in its RPAP3 domain, which binds the axonemes of cilia (Figure 2g). This is necessary for outer dynein arm attachment, and thus changes in this domain suggest functional differences which do not appear to be a result of gene duplication and subsequent divergence (King & Patel‐King, 2015). CRK‐L had decreased hydrophobicity in the Src Homology 2 (SH2) domain where tyrosine phospho‐proteins like growth factor receptors bind and decreased hydrophobicity in its SH3 domain that associates with RAC1 or RAS (Antoku & Mayer, 2009) (Figure 2d). *Anxa1* had changes in all four Annexin repeat domains, which upon Ca^2+^ binding displayed phospho‐sites (Figure 2e). Annexins activate MAPK signaling either through growth factors, or they can be directly phosphorylated by RTKs (Babbin et al., 2006). FIBP, which binds FGF1, showed fluctuations in both hydrophobicity and polarity in the annotated FIBP domain, though little is known about the mechanisms of action of this protein (Thauvin‐Robinet et al., 2016) (Figure 2f).

Another notable development gene is the homeobox transcription factor involved in segmentation and body patterning, *HoxA5* (Jeannotte et al., 2016). The protein sequence had changes in polarity and hydrophobicity near the conserved site (residues 183–188) and the beginning of the DNA‐binding homeobox domain, despite being truncated relative to *L. erinacea*. These changes suggest modified interactions between *HoxA5* and its cofactors and/or targets (Figure 2h).

To support whether changes in PSGs were specific to *P. pectinata* or shared with other sawfishes, sequencing of genomic DNA in functional domains was performed using samples from three other *Pristis* sawfishes. *Ccdc103* and *HoxA5* were chosen as they demonstrated clear chemical differences between *P. pectinata* and *L. erinacea* but were conserved enough in flanking regions of functional domains for amplification. Sequence alignments revealed that the ratios of non‐synonymous to synonymous nucleotide substitutions in both genes were lower among sawfishes than when comparing sawfishes to *L. erinacea*. At *HoxA5* conserved sites, all five substitutions were shared among sawfishes but were non‐synonymous with *L. erinacea*, with amino acid property changes at two sites (Figure 2l; Figure S3). A similar pattern was seen in *Ccdc103* among sawfishes, with changes in a functionally significant domain. In the Parp3 domain of *Ccdc103*, 18/21 changes were non‐synonymous between sawfishes compared to *L. erinacea*, and eight had an amino property change (Figure 2j; Figure S4). Further analysis of substitutions in relation to protein structure between *L. erinacea* and *P. pectinata* showed no changes in disordered domains, while all changes were found in the alpha helix structure of the RPAP3 domain (Figure S5). Fewer changes were seen among sawfishes; 10 of 13 were non‐synonymous, and seven of these included chemical changes. Given the functions of PSGs and that the ratios of non‐synonymous substitutions were lower among *Pristis* sawfishes compared to *L. erinacea* when calculated for the full‐length protein, these changes could be related to themes found in cluster 1 genes. As conservation of the biochemical changes occurred at the base of the *Pristis* branch and were conserved in all species, this leads to further credence for a role in sawfish‐specific gene function.

### 3.2 Characterization of electrosensory genes

**FIGURE 3:**
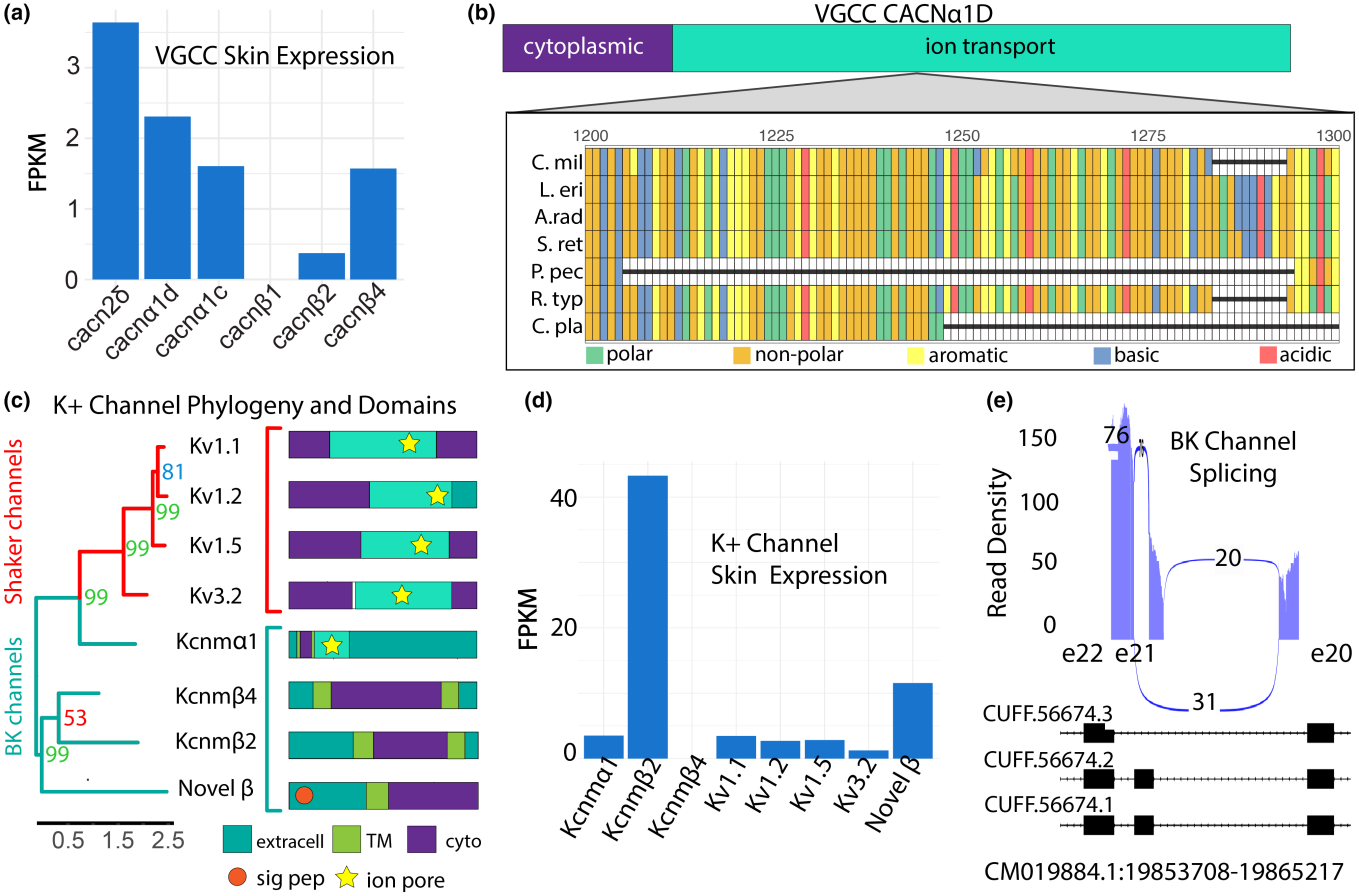
Smalltooth sawfish, *Pristis pectinata*, electroreception machinery. (a) FPKM normalized expression of voltage‐gated calcium channel α and β subunits in rostral skin. (b) Multiple sequence alignment of *Cacn*α*1D* depicting missing 91‐residue segment in *P*. *tinata* ion channel domain relative to other elasmobranchs. Species included: *Callorhinchus milii* (C. mil), *Leucoraja erinacea* (*L. e*ri), *Amblyraja radiata* (A. rad), *Scyliorhinus retifer* (S. ret), *P*. *pectinata* (P. pec), *Rhinchodon typus* (R. typ), and *Chiloscyllium plagiosum* (C. pla). Alignments are colored by amino acid chemistry. (c) Phylogenetic construction of potassium channels and subunits with bootstrap values noted (left). Sequences were aligned with ClustalW and tree was constructed by RAxML with 1000 bootstraps. Shaker channel sequences are highlighted in red, while BK channels are highlighted in light blue. Annotated protein domains of each sequence obtained using Interpro Scan (right). cyto, cytoplasm; extracell, extracellular; ion pore, ion channel pore; sig pep, signal peptide; and TM, transmembrane. (d) Normalized expression of potassium channels potentially involved electrosensing in rostral skin. Kcnm‐transcripts correspond to BK channel subunits and Kv are voltage‐gated Shaker channels. Novel β refers to the uncharacterized BK subunit found in transcriptome data. (e) GGSashimi plot of alternatively spliced BK channel transcripts in all *P. pectinata* tissues. Transcriptome expression confirms that the BK channel transcript with highest expression in the rostral skin retains exon 21 (exon 29 in *L. erinacea*).

In sawfishes, the rostrum provides an exaggerated platform for placement of the electrosensing ampullae of Lorenzini. Using the *P. pectinata* transcriptome collection described above, orthologs of all channels involved were identified to understand mediators of electrosensing in this species. First, all VGCC subunit sequences were assessed for expression in rostral skin (Figure 3a). Six VGCC‐related genes were expressed, including a *Cacnα1D* ortholog. Others were channel accessory subunits (*Cacnα2δ, Cacnb2*, and *Cacnb4*), or a *Cacnα1C*‐type VGCC, which have not been previously implicated in electrosensing in chondrichthyans. A *Cacnb2* ortholog was found in the transcriptome but was not expressed in rostral skin. Thus, as expected, the *Cacnα1D* ortholog is likely the primary mediator of electrosensing. However, *P. pectinata Cacnα1D* is missing a 91‐residue segment that would interact with intracellular effectors which is present in other rays (Figure 3b) (Bellono et al., 2017). To examine the second step in sensing, the Ca^2+^ responsive K^+^ channels were then identified, which recovered eight orthologs (Figure 3c). Clustering showed both major groups, BK (α and β), and Shaker types, were present in the gene set. A BK‐α ortholog with clear pore motif was found (*Kcnmα1*) along with three accessory BK‐β subunits, one of which was designated as novel as it could not be definitively paired with an ortholog. The four Shaker‐type channels also exhibited the requisite pore domain residues.

All eight channel components were expressed in *P. pectinata* rostral skin, except for the β‐4 ortholog (*Kcnmβ4*) (Figure 3d). Absence of this inhibitory subunit may yield sustained signaling and thereby increase the sensitivity of the electrosensory system. Further differences were seen in expression levels of β‐2 subunits. In *L. erinacea*, expression of β‐subunits in electrosensory cells is 1000‐ to 10,000‐fold lower than α‐subunit expression, yet in *P. pectinata* tissue, a BK β‐2 is expressed nearly 10‐fold to BK‐α, and the novel subunit nearly 2‐fold (Clusin et al., 2019). Another unusual observation was expression of all four shaker‐type channels, which differs from *L. erinacea* where only two, Kv1.1 and Kv1.5, have been found (Clusin et al., 2019).

Other aspects of electrosensing machinery in *P. pectinata* showed potentially divergent biology. Several insertions and deletions were found in *Kcnmα1* that may alter its function (Figure S6). In addition, retention of exon 21 (known as exon 29 in *L. erinacea*) was seen in sawfish (Figure 3e). The inclusion of this exon appears to be variable among elasmobranchs, being present in *P. pectinata*, thorny skate, *Amblyraja radiata*, and *L. erinacea* adult ampullae, but absent in *S. retifer*, whitespotted bamboo shark, *Chiloscyllium plagiosum*, and *L. erinacea* embryonic sequences (Figure S6).

## 4 DISCUSSION

Fundamental changes in developmental genes were found in *P. pectinata*, which may influence morphogenesis of the rostrum. Elongated rostral structures have evolved in chondrichthyans at least five times (Miranda‐Rottmann et al., 2010). Sawfishes (Pristidae) and sawsharks (Pristiophoridae) are the only extant families that possess toothed rostra, but similar structures are also found in the fossil record, including two species of chimaeras, *Squaloraja polyspondyla* and *Acanthorhina jaekel* (Holocephali), Sclerorhynchoidei (Rajiformes), and *Bandringa rayi* (Elasmobranchii) (Miranda‐Rottmann et al., 2010; Sallan & Coates, 2014). Convergent evolution of elongated rostral structures suggests an evolutionary advantage in prey detection, likely through heightened bioelectric sensing, which may provide sensory information at night, at depth, or in turbid coastal waters (Wueringer, 2012). The cohort of genes uniquely under selection in *P. pectinata* may provide a framework for the genetic changes that underpin the morphogenetic origins of saw‐like rostral structures. Primarily, this appears to be through reshaping the signaling environment, as was observed in this study, with changes in FGF/MAPK mediators. Simultaneously, positive selection was also seen in *HoxA5*, a transcription factor that establishes regions along the dorsoventral axis. However, in vertebrate development, cluster 5 Hox genes are typically expressed in the somites which eventually become the upper thorax and organs, thus changes in this gene in *P. pectinata* may be related to differences in pectoral or synarcual morphology, and not related to the rostrum.

Several *P. pectinata* PSGs are known to interact with marine pollutants including polycyclic aromatic hydrocarbons (PAHs), heavy metals, bisphenol‐A's (BPAs), brominated flame retardants (BFRs), and polychlorinated benzodioxins (PCBs) (Davis et al., 2021). For example, *Crk‐l* expression is affected by BPAs, BFRs, and PCBs, and *Anxa1* is upregulated in response to PAH exposure in sea turtles as a response to increased production of reactive oxygen species (Cocci et al., 2019). As predators, heavy metals, PCBs, and other persistent organic pollutants can bioaccumulate in elasmobranch tissues, potentially disrupting critical processes such as metabolism, immune function, and reproduction, although pollutant levels in sawfish tissues have not been reported (Hamers et al., 2006; Martins et al., 2021; Tiktak et al., 2020). Characterizing how sawfish pollutant‐interacting genes may be differentially affected by these compounds could shed light on toxicological risks and may help identify biomarkers for pollutant‐induced stress (Cullen et al., 2019).

A top conservation priority for all sawfishes is to reduce injuries and mortalities in fisheries, especially trawl fisheries (Poulakis & Grubbs, 2019). Understanding the basis of sawfish electrosensing at a molecular level may support the development of more effective deterrent technologies that exploit this sensing modality. In future studies, molecular‐level responses to stimuli, such as electric fields, should be studied to refine optimal experimental conditions, and used in parallel with aquarium trials to elicit avoidance responses (Abrantes et al., 2021). This study found multiple novel differences in genes associated with electroreception of sawfish relative to other elasmobranchs. *Pristis pectinata Cacnα1D* has lost a 91‐residue motif, which also is variable in other chondrichthyans such as *C. milii* and the whale shark, *Rhincodon typus*, indicating that it is a site of functional novelty (Figure 3b) (Bellono et al., 2017). Of the four Shaker‐type channels, two have been observed in *L. erinacea*, but have only been demonstrated to work in conjunction with *Cacnα1D* orthologs in ampullae of *S. retifer* (Bellono et al., 2017; Clusin et al., 2019). Together, these results suggest that substantial changes in the cellular machinery that could be involved in the initial events used by *P. pectinata* to detect the bioelectric signals of prey.

Analysis of electrosensory genes revealed numerous differences in functional domains and expression of channels and subunits which were previously not implicated in electrosensing in chondrichthyans. As batoids diverged significantly from *L. erinacea*, sawfishes appear to possess unique electrosensing mechanisms that leverage K^+^ channels found in sharks, in addition to those described in the model skate species. Considering skate‐type electrosensing alone, elevated expression of multiple previously uncharacterized subunits suggests *P. pectinata* may have substantially altered BK channel physiology. The ion pore‐containing BK‐α subunits can differentially associate with β‐subunits to modulate membrane repolarization to alter activation rates or affect calcium sensitivity, drawing into question the role of the novel β‐subunit in the overall performance of the system (Brenner et al., 2000; Gonzalez‐Perez & Lingle, 2019; Li & Yan, 2016). In addition, alternative splicing at exon 21 has been identified in BK channel ampullary transcripts of *L. erinacea* and in auditory hair cells of chick cochlea, though the effect on electrosensing is not known (King et al., 2015).

Altogether, these results suggest unique physiology of ion channels and subunits in *P. pectinata*, and their identification could allow more effective bycatch‐reduction technology. Developing this technology for trawls is a high conservation priority, as shrimp trawls have been identified as having the highest bycatch risk for sawfishes in the ‘stronghold’ nations of the U.S. and Australia (Abrantes et al., 2021; Brewer et al., 2006; Graham et al., 2022).

In addition to providing an annotation of the publicly available genome, this study highlights the value of genomic approaches in conservation efforts, particularly through the identification and characterization of electrosensory genes and subunits, and genes that could ultimately be used to reduce incidence of bycatch. Global recovery of sawfishes requires aggressive conservation planning that could benefit from novel methodological approaches to support modern, high‐tech solutions. Results from this work also suggest that the impacts of pollutants need to be more deeply investigated and that physiological responses may differ between sawfishes and existing model organisms. These data will also support the ability to build experimental systems to test Ca^2+^ channel behavior in a controlled in vitro setting that can be used to assess and quantify the performance of sawfish electrosensing, facilitating the development of behavior‐modifying technology. Together, these data and insights provide the foundation to support key future research, with the goal of supporting global recovery of imperiled sawfishes.

## AUTHOR CONTRIBUTIONS

**Taiya M. Jarva:** Data curation (equal); formal analysis (equal); methodology (equal); visualization (equal); writing – original draft (equal); writing – review and editing (equal). **Nicole M. Phillips:** Conceptualization (equal); data curation (equal); funding acquisition (equal); project administration (equal); supervision (equal); writing – original draft (equal); writing – review and editing (equal). **Cory Von Eiff:** Formal analysis (equal). **Gregg R. Poulakis:** Resources (equal); writing – review and editing (equal). **Gavin Naylor:** Resources (equal); writing – review and editing (equal). **Kevin A. Feldheim:** Conceptualization (equal); project administration (equal); resources (equal); writing – review and editing (equal). **Alex S. Flynt:** Conceptualization (equal); formal analysis (equal); funding acquisition (equal); investigation (equal); project administration (equal); resources (equal); supervision (equal); visualization (equal); writing – original draft (equal); writing – review and editing (equal).

## CONFLICT OF INTEREST STATEMENT

The authors declare no conflicts of interest.

## Supporting information


Appendix S1

Appendix S2

Figure S1

Figure S2

Figure S3

Figure S4

Figure S5

Figure S6

Tables S1–S3

## Data Availability

Raw sequence data used to assemble the transcriptome can be found under NCBI accession PRJNA864825. The transcriptome assembly described in this paper has been deposited in DDBJ/EMBL/GenBank under Accession #GKOB01000000. Code used to perform custom analyses can be found at https://github.com/stupornova33/sawfish_code.
