# Supplementary material for: Gene expression, evolution, and the genetics of electrosensing in the smalltooth sawfish, Pristis pectinata
Source: Ecol Evol. 2024 Apr 29;14(5):e11260. doi: 10.1002/ece3.11260 (PMC11057056; doi:10.1002/ece3.11260)
Supplement: Supplementary file 1 — Appendix S1 [file ECE3-14-e11260-s003.zip › Jarva_etal_Sup_info_revised_final.docx]

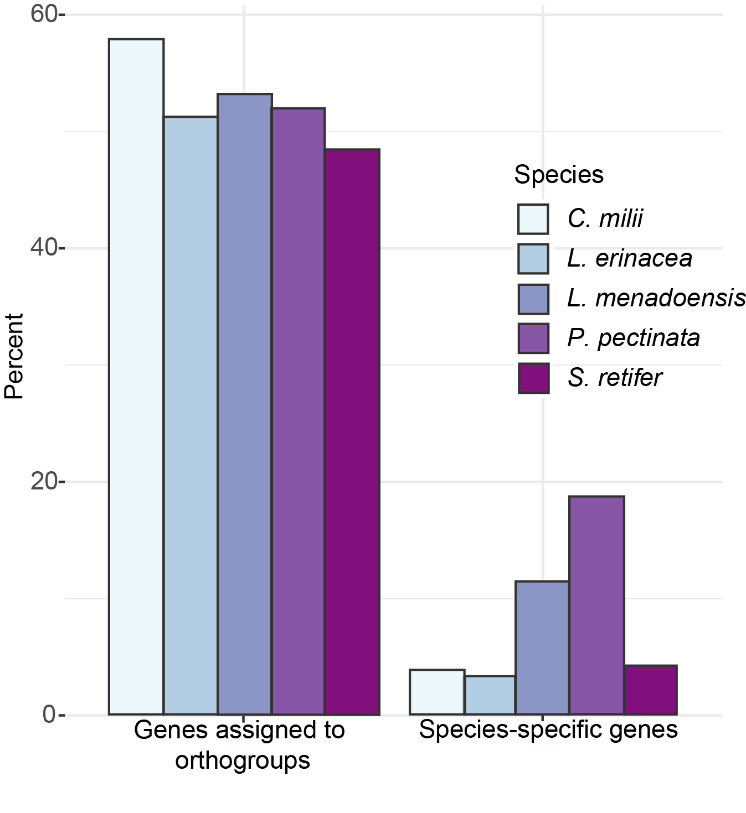


**Supplementary Figure 1.** Left: Percentage of genes assigned to orthogroups by OrthoFinder. Right: Percentage of genes unique to each species. Species included: chain catshark, *Scyliorhinus retifer*, Indonesian coelacanth, *Latimeria menadoensis*, Australian ghostshark, *Callorhinchus milii*, smalltooth sawfish, *Pristis pectinata*, and little skate, *Leucoraja erinacea*.


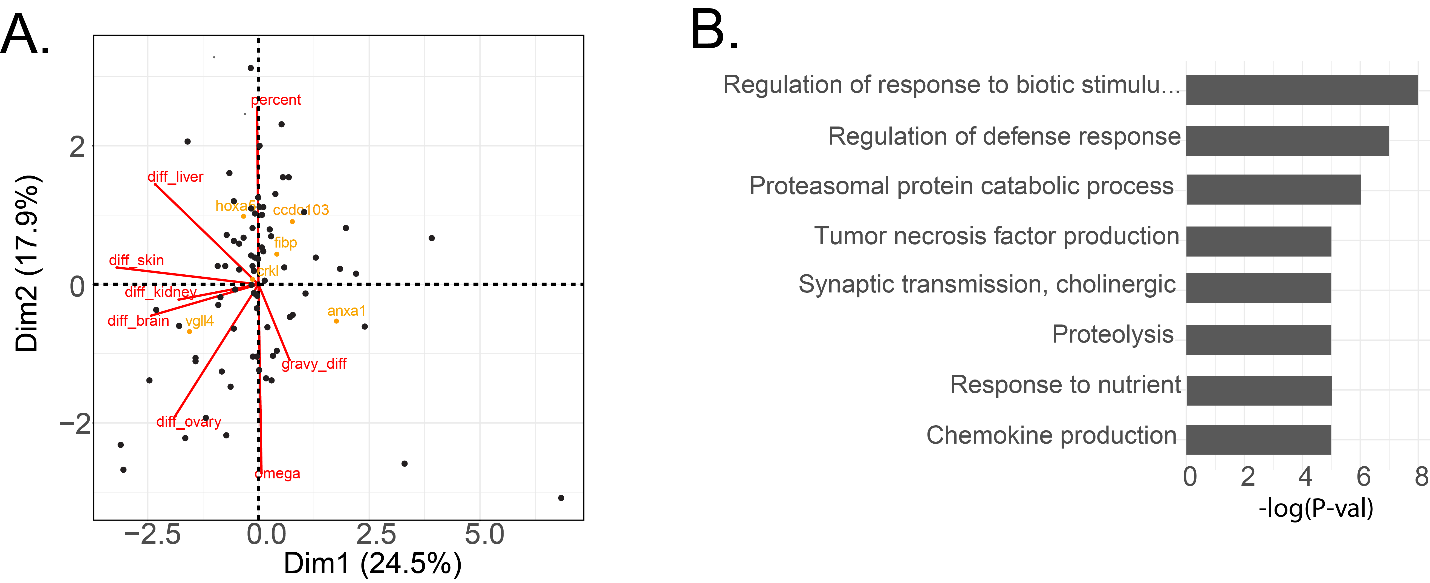


**Supplementary Figure 2. Smalltooth sawfish, *Pristis pectinata*, genes under selection. A)** PCA of genes under selection using omega value, percent of sites, change in GRAVY value relative to little skate, *Leucoraja erinacea*, and change in expression relative to chain catshark, *Scyliorhinus retifer*. Genes of interest from Cluster 1 are shown in yellow, variables are shown in red. **B)** Top 8 most enriched gene ontology terms related to biological processes from genes grouped into Cluster 2 by PCA analysis plotted by -log(p-value).


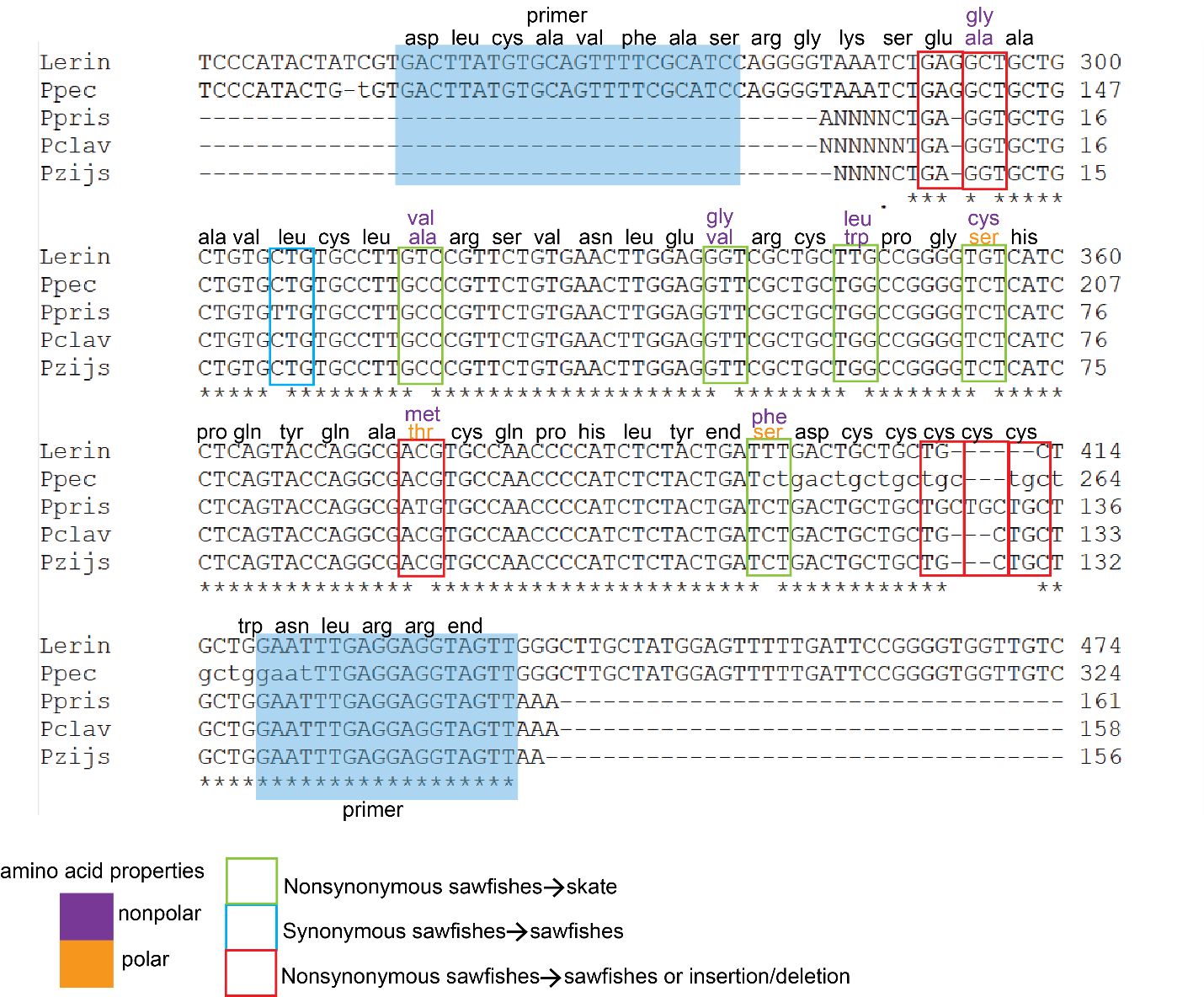


**
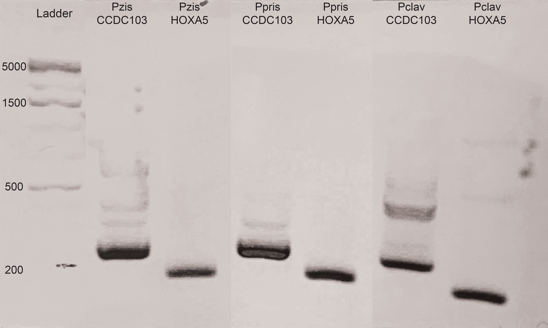
**

**Supplementary Figure 3**

Annotation of substitutions in *HoxA5* between dwarf, green, largetooth, and smalltooth sawfishes (*Pristis clavata*, *P. zijsron*, *P. pristis*, *P. pectinata*, respectively) and little skate, *Leucoraja erinacea*. Changes in amino acids are noted above aligned nucleotide sequences. Primer sequences are shaded in blue. Gel showing amplicons from different species below


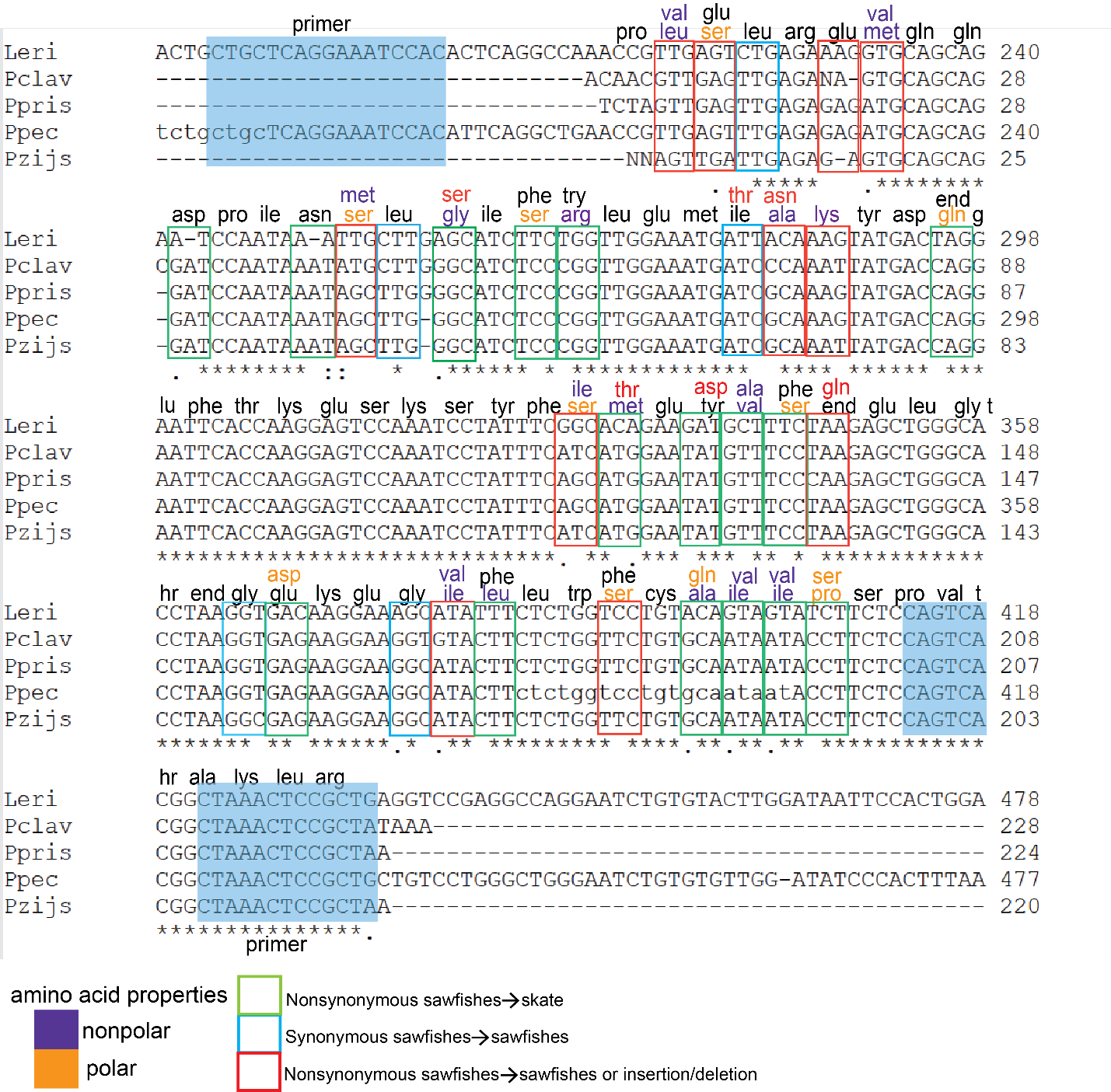


**Supplementary Figure 4**

Annotation of substitutions in *Ccdc103* between dwarf, green, largetooth, and smalltooth sawfishes (*Pristis clavata*, *P. zijsron*, *P. pristis*, *P. pectinata*, respectively) and little skate, *Leucoraja erinacea*. Changes in amino acids are noted above aligned nucleotide sequences. Primer sequences are shaded in blue.


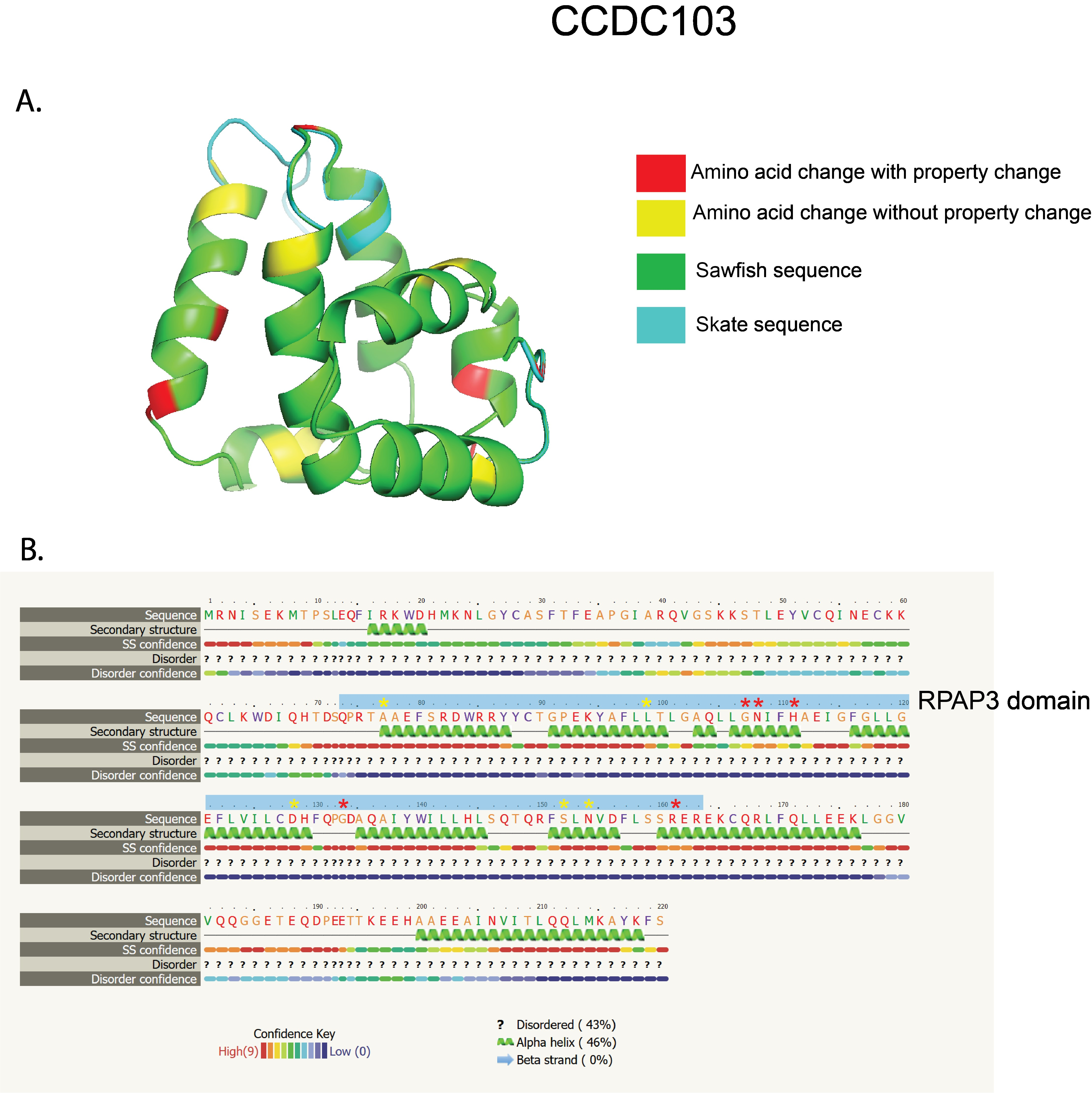


**Supplementary Figure 5.**

Analysis of protein structure of CCDC103. **A)** Phyre2-predicted structures of CCDC103 in skate (*L. erinacea*) and smalltooth sawfish (*P. pectinata*) aligned and highlighted by PyMOL (Kelley et al., 2015; Schrödinger & Warren Delano, 2020). **B)** Sequence, secondary structure prediction, and confidence levels of predicted structure by Phyre2.


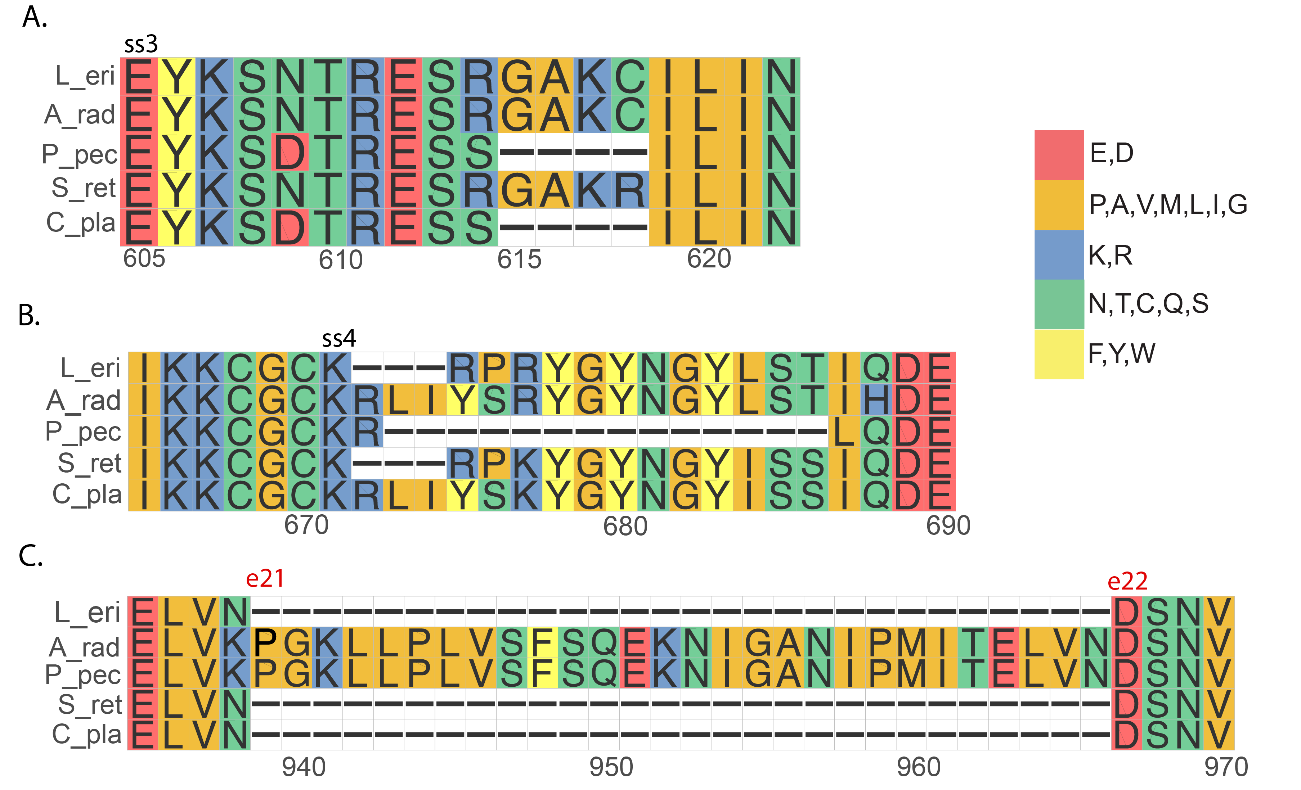


**Supplementary Figure 6**

Insertions, deletions, and splice sites identified in *Pristis pectinata* (P_pec) BK alpha K^+^ channel relative to *Leuroraja erinacea* (L_eri), *Amblyraja radiata* (A_rad), *Scyliorhinus retifer* (S_ret) and *Chiloscyllium plagiosum* (C_pla). Ss = splice site. e = exon. Plot colors indicate amino acid chemical properties. **A)** splice site 3. **B)** splice site 4. **C)** exon 21.

| Tissue | Concentration (ng/ul) | Purity (A260/A280) | RIN |
| --- | --- | --- | --- |
| Brain | 174.7 | 1.83 | 6.7 |
| Liver | 962.4 | 2.01 | 6.9 |
| Ovary | 1660.7 | 2.03 | 7.0 |
| Kidney | 1150.0 | 2.04 | 7.3 |
| Skin | 273.9 | 1.98 | 7.5 |

**Supplementary Table 1.** Concentrations and purities of smalltooth sawfish, *Pristis pectinata*, RNA samples by tissue obtained by nanodrop and Bioanalyzer. For RNA, chemical purity is indicated by A260/A280 of 2.0. RNA integrity number (RIN) ranges from 1 to 10, where 10 is intact and 1 is degraded.

|  | Smalltooth sawfish, Pristis pectinata | Chain catshark Scyliorhinus retifer | Little skate, Leucoraja erinacea | Indonesian coelacanth, Latimeria menadoensis, | Australian ghostshark, Callorhinchus milii |
| --- | --- | --- | --- | --- | --- |
| No. transcripts in dataset | 175,569 | 107,231 | 103,996 | 66,138 | 92,334 |
| Accession | PRJNA864825 | GEO: GSM643958 | GEO: GSM643957 | GAPS01066138 | GEO: GSM643959 |
| %BUSCO complete | 89.47% C  3.87% F | 56.6% C  22.1% F | 60.4% C  20.4% F | 40.9% C  18.9% F | 47.3% C  26.9% F |

**Supplementary Table 2.** Number of transcripts, NCBI accession numbers, and percentage of complete (C) and fragmented (F) orthologs via BUSCO analysis of transcriptomes for all taxa used in positive selection analyses.

| Species | Unique genes | Total genes |
| --- | --- | --- |
| Smalltooth sawfish, *Pristis pectinata* | 79 | 96 |
| Chain catshark*, Scyliorhinus retifer* | 26 | 38 |
| Australian ghostshark*, Callorhinchus milii* | 76 | 95 |
| Little skate, *Leucoraja erinacea* | 40 | 51 |
| Indonesian coelacanth, *Latimeria menadoensis* | 49 | 60 |

**Supplementary Table 3.** Number of unique genes and total number of genes found under selection in aBSREL analysis by species.

| Sample | Accession code | Run |
| --- | --- | --- |
| Adult 1 kidney | SAMD00098998 | DRR111789 |
| Adult 1 medulla | SAMD00098989 | DRR111780 |
| Adult 1 ovary | SAMD00098994 | DRR111785 |
| Adult 1 liver | SAMD00098997 | DRR111788 |
| Adult 3 skin | SAMD00099043 | DRR111834 |

**Supplementary Table 4.** NCBI sample names, accession codes, and run identifiers for the chain catshark, *Scyliorhinus retifer,* expression data used in PCA analysis.

| Sample | Total reads | Unaligned | Aligned once | Multimapping | Overall rate |
| --- | --- | --- | --- | --- | --- |
| A1 Kidney | 10208584 | 99.41% | 0.58% | 0.01% | 0.59% |
| A1 Medulla | 8936204 | 99.32% | 0.67% | 0.01% | 0.68% |
| A1 Ovary | 8892600 | 99.30% | 0.69% | 0.01% | 0.70% |
| A1 Liver | 7162306 | 99.61% | 0.38% | 0.01% | 0.39% |
| A3 Skin | 9125803 | 99.35% | 0.63% | 0.02% | 0.65% |

**Supplementary Table 5.** Number and percent of reads mapped to genes orthologous to the smalltooth sawfish, *Pristis pectinata*, PSGs per sample for the chain catshark, *Scyliorhinus retifer*, expression data used in PCA analysis.

| gene | nsyn | syn | ISS pt1 | ISS.c pt1 | ISS < ISS.c | ISS.c pt2 | ISS < ISS.c pt2 |
| --- | --- | --- | --- | --- | --- | --- | --- |
| mad2l1bp | 68.24751465 | 0.015342834 | 0.2979 | 0.8034 | Little saturation | 0.7892 | Little saturation |
| ube2k | 27.71654379 | 0.015864337 | 0.0765 | 0.7526 | Little saturation | 0.6797 | Little saturation |
| ube2g1 | 31.30747885 | 0.015371404 | 0.0921 | 0.719 | Little saturation | 0.5946 | Little saturation |
| tmod3 | 7.740042863 | 0.0147169 | 0.2251 | 0.7803 | Little saturation | 0.7272 | Little saturation |
| tmem115 | 0.175362613 | 4.0841E-05 | 0.1956 | 0.7685 | Little saturation | 0.695 | Little saturation |
| tmem70 | 0.175219408 | 4.0803E-05 | 0.2859 | 0.7486 | Little saturation | 0.6785 | Little saturation |
| tirap | 1.43615889 | 0.000120479 | 0.3594 | 0.7503 | Little saturation | 0.6787 | Little saturation |
| stard9 | 133.3724232 | 0.02372274 | 0.3315 | 0.7869 | Little saturation | 0.7851 | Little saturation |
| slc46a1 | 20.6888195 | 0.002664867 | 0.3584 | 0.759 | Little saturation | 0.6842 | Little saturation |
| sept2 | 519.256961 | 0.038144319 | 0.1417 | 0.7544 | Little saturation | 0.6431 | Little saturation |
| sec62 | 0.713132866 | 0.033312161 | 0.3835 | 0.7832 | Little saturation | 0.7305 | Little saturation |
| mfap3 | 145.9950837 | 0.003378052 | 0.3949 | 0.7595 | Little saturation | 0.649 | Little saturation |
| hm13 | 70.17835227 | 0.023876108 | 0.3557 | 0.7889 | Little saturation | 0.7367 | Little saturation |
| gps1 | 12.30100156 | 0.010408471 | 0.2799 | 0.8014 | Little saturation | 0.7493 | Little saturation |
| c1orf52 | 1106.885301 | 0.013149186 | 0.3303 | 0.7622 | Little saturation | 0.7192 | Little saturation |
| bckdhb | 82.60212403 | 0.017071841 | 0.2655 | 0.7881 | Little saturation | 0.7359 | Little saturation |
| atp6v1g1 | 21.68743298 | 0.003870761 | 0.1889 | 0.758 | Little saturation | 0.7172 | Little saturation |
| apitd1 | 63.78277863 | 0.01372788 | 0.4742 | 0.7588 | Little saturation | 0.7132 | Little saturation |
| ambp | 33.15613451 | 0.03631689 | 0.3954 | 0.7685 | Little saturation | 0.7488 | Little saturation |
| chrna1 | 987.3243756 | 0.037430086 | 0.3116 | 0.7607 | Little saturation | 0.7293 | Little saturation |
| mfsd12 | 28.9633664 | 0.003964744 | 0.4458 | 0.7693 | Little saturation | 0.7151 | Little saturation |
| parp3 | 6.032603784 | 0.050719673 | 0.4559 | 0.7851 | Little saturation | 0.7327 | Little saturation |
| sass6 | 282.8415783 | 0.025569125 | 0.4376 | 0.7724 | Little saturation | 0.757 | Little saturation |
| rnmtl1 | 3.898682418 | 0.008234327 | 0.2335 | 0.758 | Little saturation | 0.7175 | Little saturation |
| anxa1 | 207.5144955 | 0.018032195 | 0.2666 | 0.7632 | Little saturation | 0.712 | Little saturation |
| ccdc103 | 121.1825273 | 0.00798693 | 0.4404 | 0.7545 | Little saturation | 0.6809 | Little saturation |
| fibp | 5.818441714 | 0.104204016 | 0.2042 | 0.7881 | Little saturation | 0.7359 | Little saturation |
| hoxa1 | 1.767833678 | 0.007212297 | 0.1511 | 0.7668 | Little saturation | 0.7135 | Little saturation |

**Supplementary Table 6.** DAMBE results from genes with dN/dS > 10000. “ISS pt1 < ISS.c pt1 indicates “Little saturation” for a symmetric tree, while ISS < ISS.c pt2 indicates "Little saturation” for an unlikely asymmetric tree. ISS > ISS.c would indicate substantial substitution saturation (Xuhua Xia & P Lemey, 2009).

**Supplementary References**

Kelley, L. A., Mezulis, S., Yates, C. M., Wass, M. N., & Sternberg, M. J. E. (2015). The Phyre2 web portal for protein modeling, prediction and analysis. *Nature Protocols*, *10*(6), 845–858. https://doi.org/10.1038/nprot.2015.053

Schrödinger, L., & Warren Delano. (2020). *The PyMOL Molecular Graphics System* (2.0).

Xuhua Xia, & P Lemey. (2009). *The Phylogenetic Handbook*.
