# Supplementary figures and images for: Gene expression, evolution, and the genetics of electrosensing in the smalltooth sawfish, Pristis pectinata
Source: Ecol Evol. 2024 Apr 29;14(5):e11260. doi: 10.1002/ece3.11260 (PMC11057056; doi:10.1002/ece3.11260)

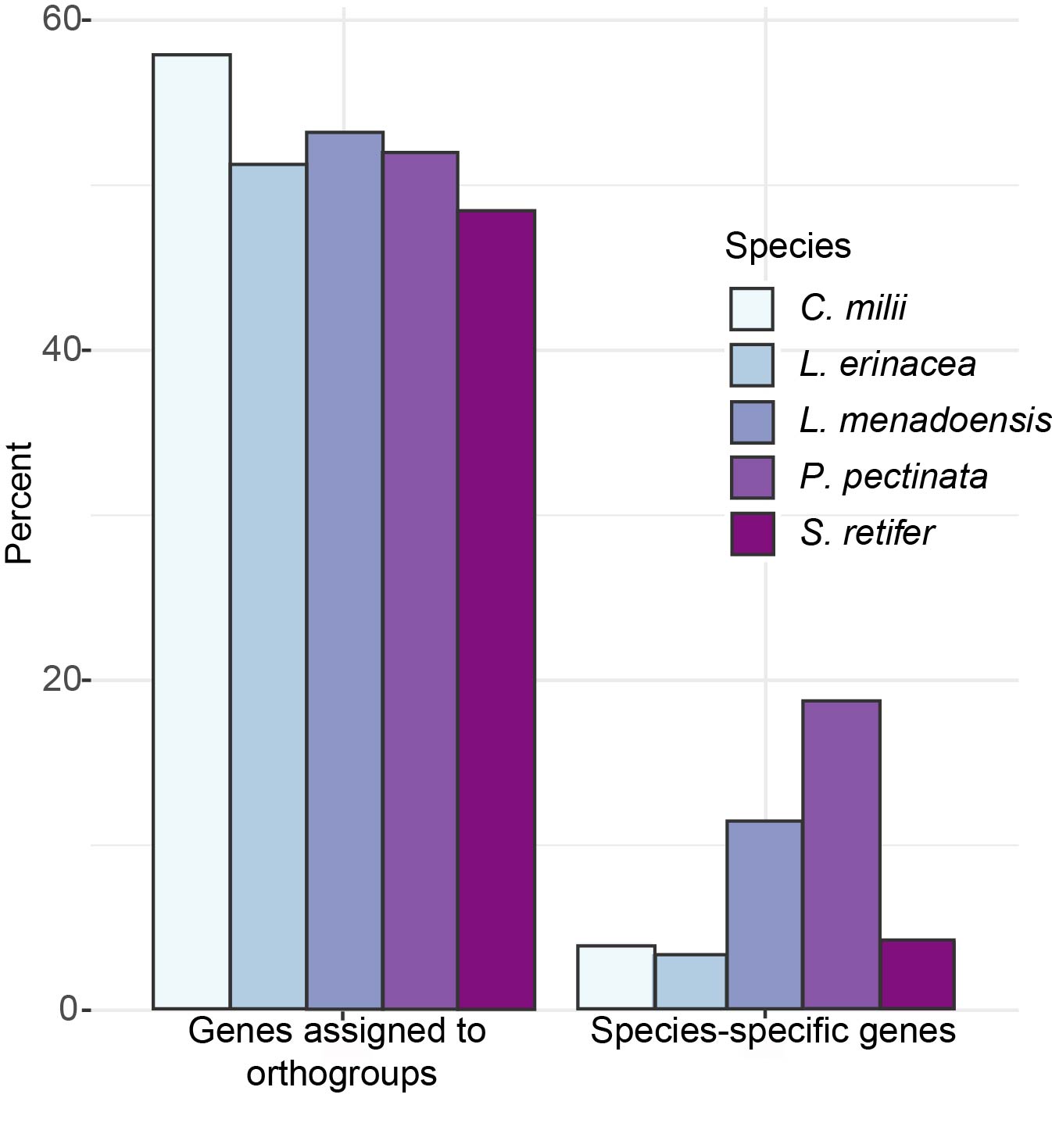

Supplement: Supplementary file 3 — Figure S1 [file ECE3-14-e11260-s002.jpg]

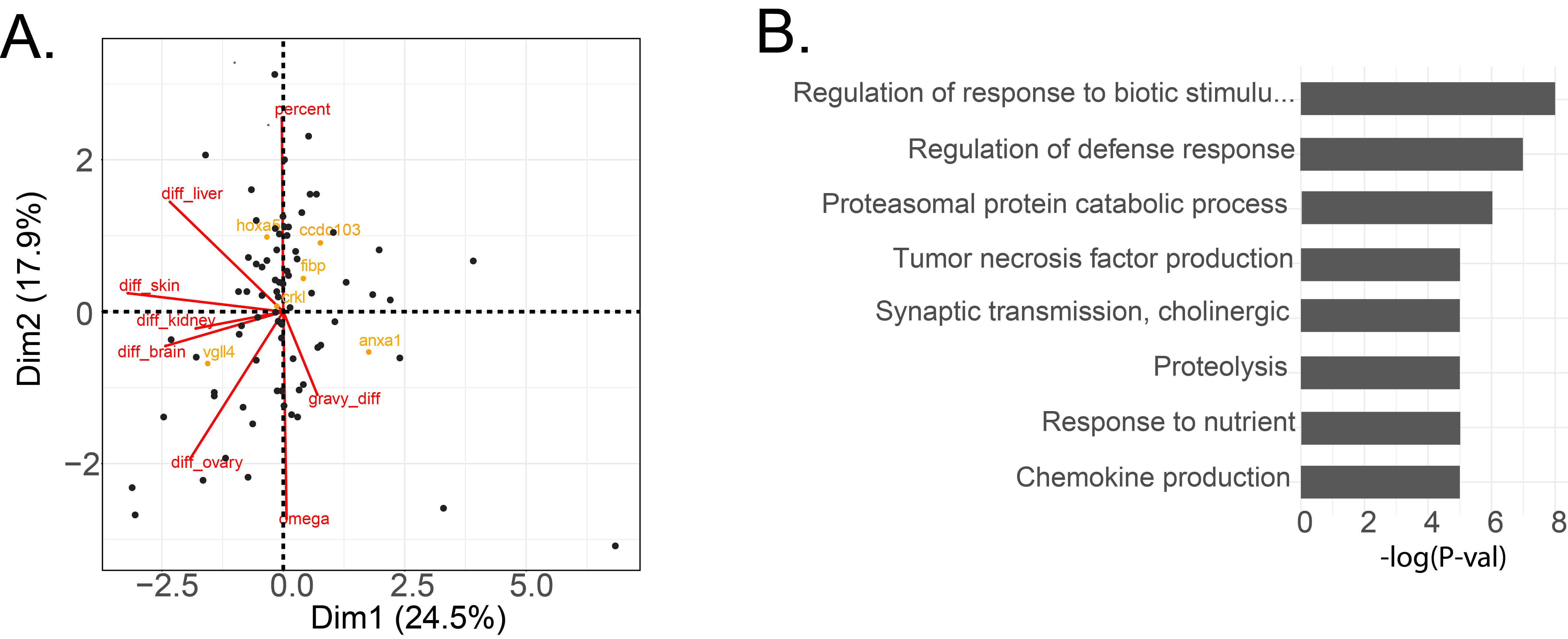

Supplement: Supplementary file 4 — Figure S2 [file ECE3-14-e11260-s009.jpg]

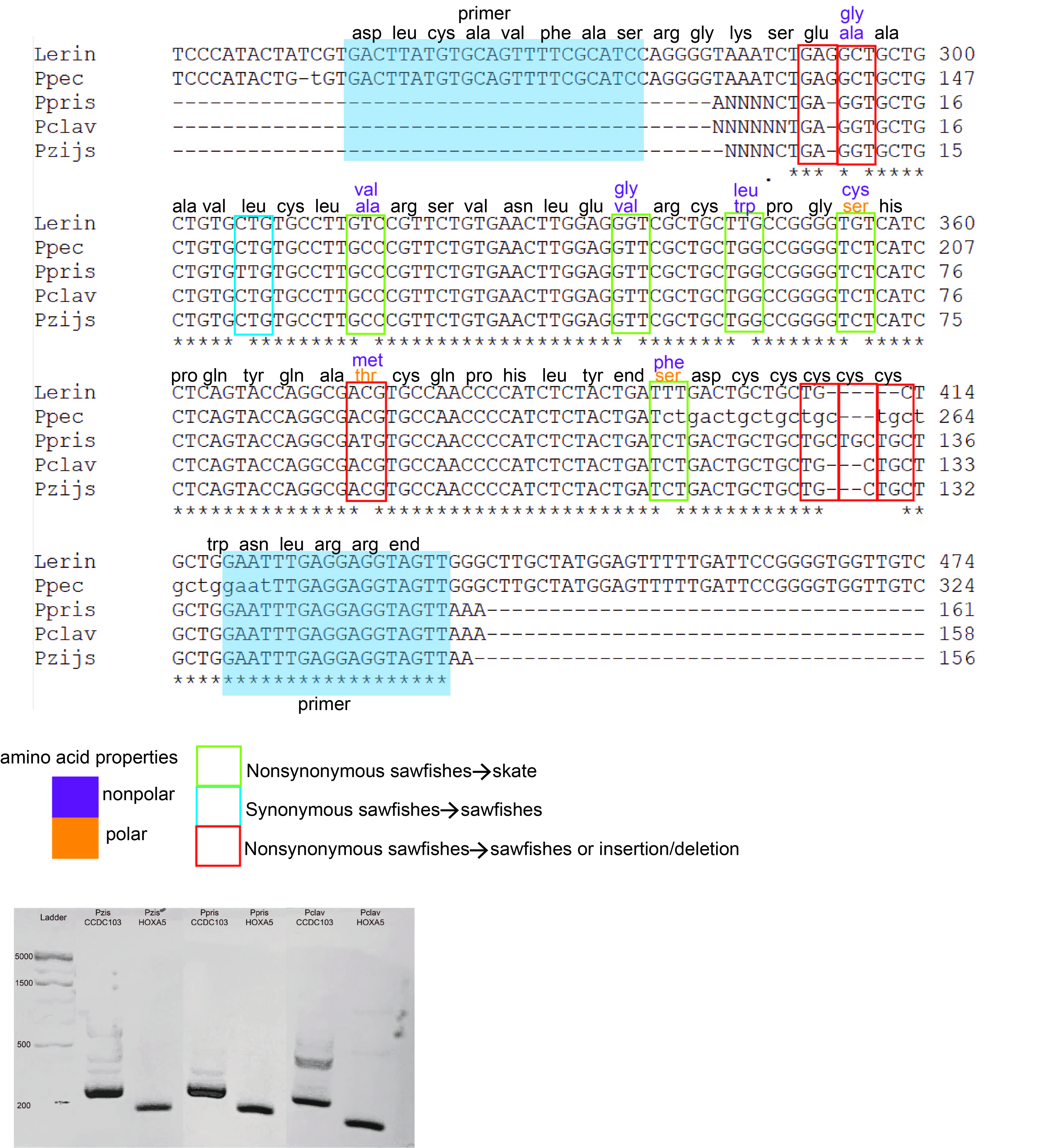

Supplement: Supplementary file 5 — Figure S3 [file ECE3-14-e11260-s007.jpg]

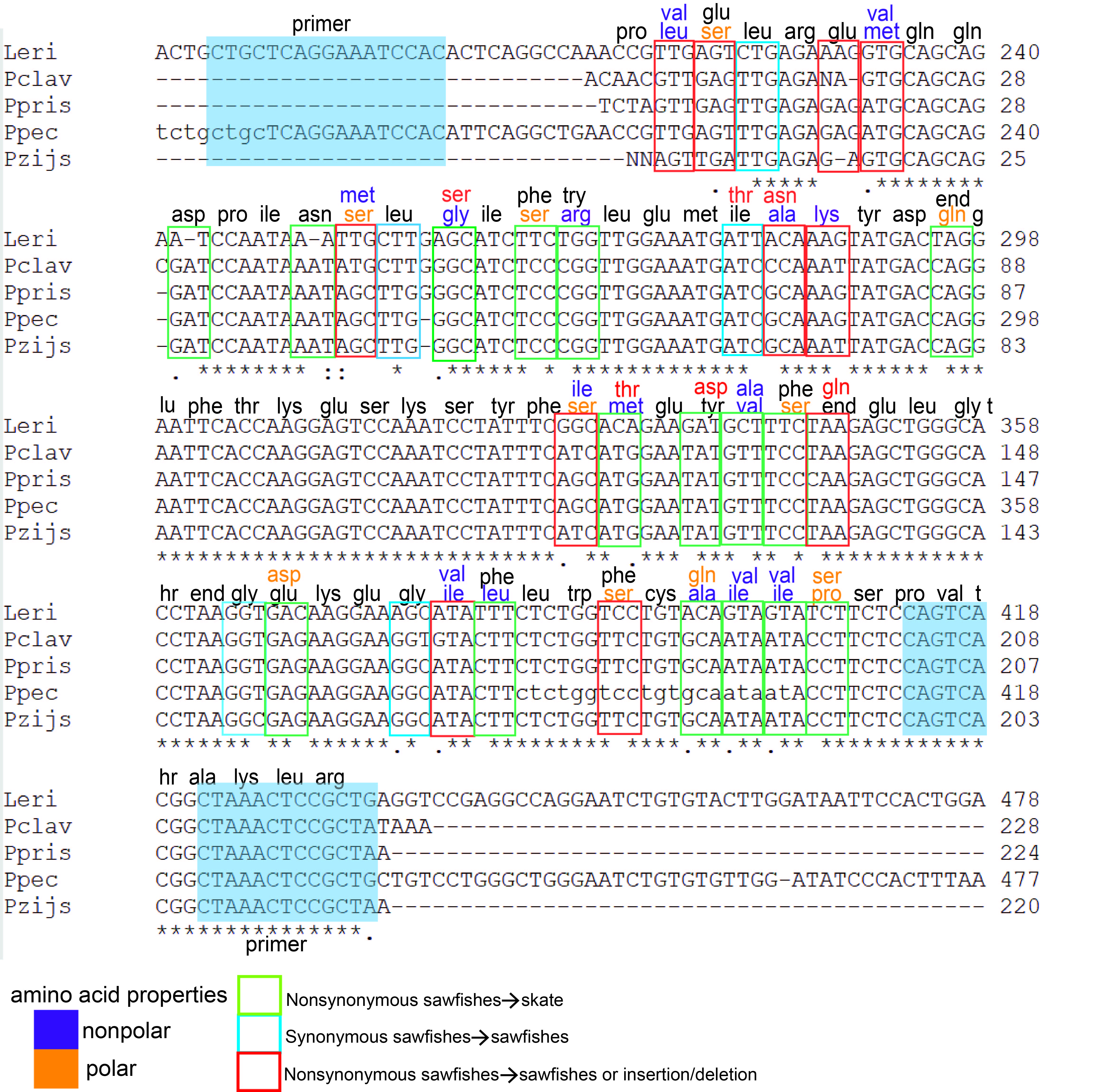

Supplement: Supplementary file 6 — Figure S4 [file ECE3-14-e11260-s005.jpg]

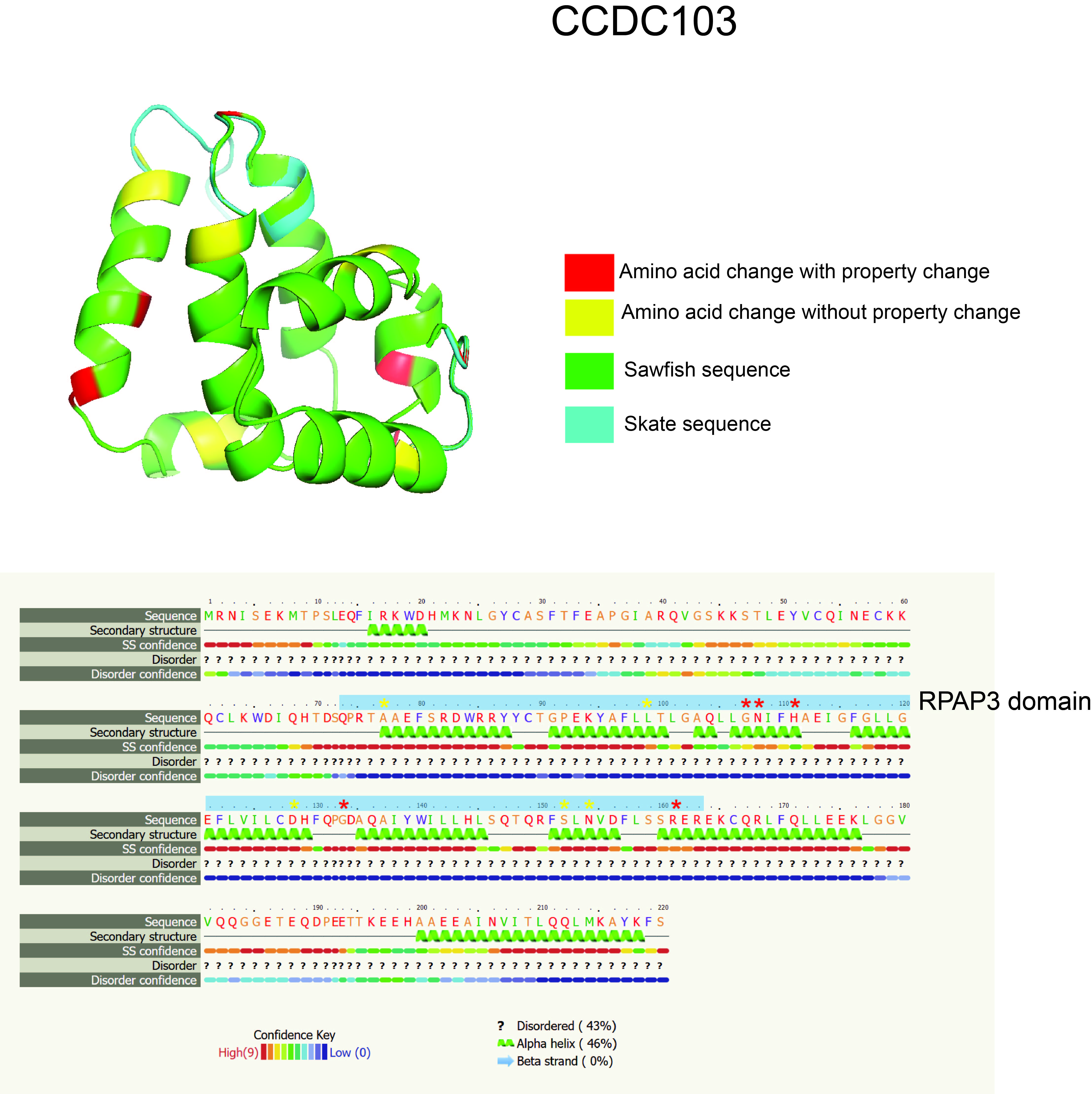

Supplement: Supplementary file 7 — Figure S5 [file ECE3-14-e11260-s004.jpg]

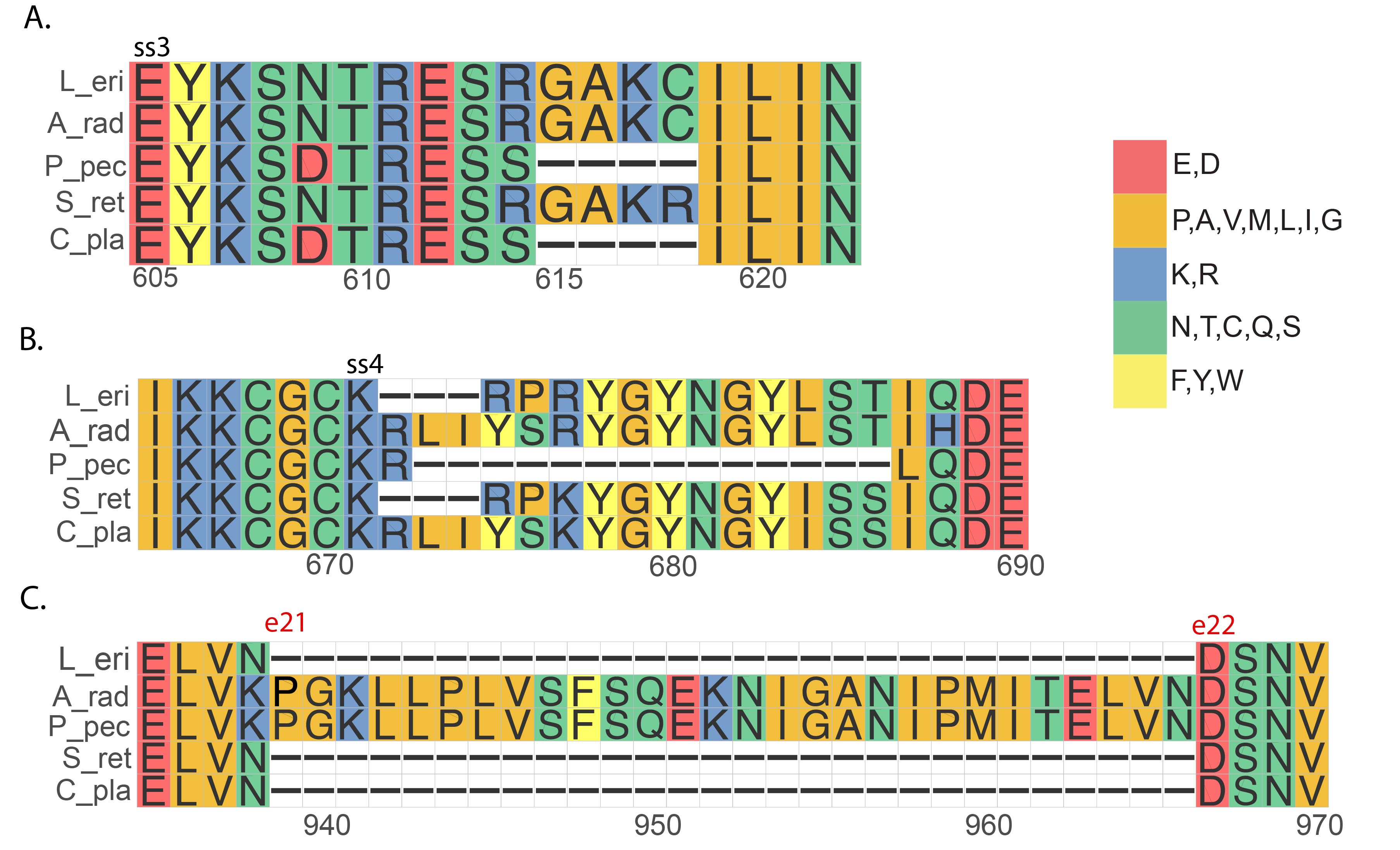

Supplement: Supplementary file 8 — Figure S6 [file ECE3-14-e11260-s008.jpg]
