# Supplementary material for: Gene expression, evolution, and the genetics of electrosensing in the smalltooth sawfish, Pristis pectinata
Source: Ecol Evol. 2024 Apr 29;14(5):e11260. doi: 10.1002/ece3.11260 (PMC11057056; doi:10.1002/ece3.11260)
Supplement: Supplementary file 9 — Tables S1–S3 [file ECE3-14-e11260-s001.docx]

| Tissue | Concentration (ng/ul) | Purity (A260/A280) | RIN |
| --- | --- | --- | --- |
| Brain | 174.7 | 1.83 | 6.7 |
| Liver | 962.4 | 2.01 | 6.9 |
| Ovary | 1660.7 | 2.03 | 7.0 |
| Kidney | 1150.0 | 2.04 | 7.3 |
| Skin | 273.9 | 1.98 | 7.5 |

**Supplementary Table 1.** Concentrations and purities of smalltooth sawfish, *Pristis pectinata*, RNA samples by tissue obtained by nanodrop and Bioanalyzer. For RNA, chemical purity is indicated by A260/A280 of 2.0. RNA integrity number (RIN) ranges from 1 to 10, where 10 is intact and 1 is degraded.

|  | Smalltooth sawfish, Pristis pectinata | Chain catshark Scyliorhinus retifer | Little skate, Leucoraja erinacea | Indonesian coelacanth, Latimeria menadoensis, | Australian ghostshark, Callorhinchus milii |
| --- | --- | --- | --- | --- | --- |
| No. transcripts in dataset | 175,569 | 107,231 | 103,996 | 66,138 | 92,334 |
| Accession | PRJNA864825 | GEO: GSM643958 | GEO: GSM643957 | GAPS01066138 | GEO: GSM643959 |
| %BUSCO complete | 89.47% C  3.87% F | 56.6% C  22.1% F | 60.4% C  20.4% F | 40.9% C  18.9% F | 47.3% C  26.9% F |

**Supplementary Table 2.** Number of transcripts, NCBI accession numbers, and percentage of complete (C) and fragmented (F) orthologs via BUSCO analysis of transcriptomes for all taxa used in positive selection analyses.

| Species | Unique genes | Total genes |
| --- | --- | --- |
| Smalltooth sawfish, *Pristis pectinata* | 79 | 96 |
| Chain catshark*, Scyliorhinus retifer* | 26 | 38 |
| Australian ghostshark*, Callorhinchus milii* | 76 | 95 |
| Little skate, *Leucoraja erinacea* | 40 | 51 |
| Indonesian coelacanth, *Latimeria menadoensis* | 49 | 60 |

**Supplementary Table 3.** Number of unique genes and total number of genes found under selection in aBSREL analysis by species.
